# The CgATG16 Was Involved in Growth, Development and Virulence Through Autophagy Modulation in the Rubber Tree Anthracnose Fungus *Colletotrichum gloeosporioides*

**DOI:** 10.3390/jof11120828

**Published:** 2025-11-23

**Authors:** Haoran Cheng, Zhirui Huang, Jiajia Xie, Erxiu He, Qiannan Wang, Bang An, Chaozu He, Hongli Luo

**Affiliations:** 1 Sanya Nanfan Research Institute of Hainan University, School of Tropical Agriculture and Forestry, Hainan University, Sanya 572025, China; chenghaoran0511@163.com (H.C.); 17837109720@163.com (Z.H.); jxs1528554358@163.com (J.X.); erxiuhe@163.com (E.H.); wangqiannan@hainanu.edu.cn (Q.W.); anbang@hainanu.edu.cn (B.A.); czhe@hainanu.edu.cn (C.H.); 2 Hainan Yazhou Bay Seed Laboratory, Sanya 572025, China; 3 Henan Academy of Agricultural Sciences, Zhengzhou 451451, China

**Keywords:** *Colletotrichum gloeosporioides*, ATG16, pathogenicity, autophagy

## Abstract

*Colletotrichum gloeosporioides* causes rubber tree anthracnose and leads to serious loss in natural rubber production. Autophagy is a highly conserved process to maintain nutrient recycling and plays important roles in growth, development and pathogenicity in plant pathogenic fungi. The process of autophagy is modulated by a series of autophagy-related (ATG) genes. ATG16 is a subunit of the ATG12-ATG5-ATG16 complex which functions in a manner analogous to an E3-like enzyme which is essential for autophagosome formation. However, the function of the ATG16 homolog in *C. gloeosporioides* remains unknown. In this study, the ATG16 homolog of *C. gloeosporioides* was identified and named as CgATG16. The expression level of *CgATG16* was particularly higher in conidium, germination, appressorium, and the early stage of infection, and significantly induced by nutritional deficiency. Absence of *CgATG16* led to slower colony growth, decreased conidia production and germination rate, longer germ tube cells, lower appressorium formation rate and impaired pathogenicity to rubber tree leaves. Absence of *CgATG16* resulted in lower melanin content with decreased expression of polyketide synthase gene *CgPKS1* and scytalone dehydratase gene *CgSCD1*. Moreover, absence of *CgATG16* also led to the universal autophagy marker ATG8-GFP failing to enter into the vacuoles in mycelium and during appressorium development with a significantly reduced autophagosome number. Both rapamycin and cyclic adenosine monophosphate (cAMP) partially restored the appressorium formation ability in *CgATG16* knockout mutant. Absence of *CgATG16* increased the activity of target of rapamycin (TOR) kinase and decreased the content of cAMP. These data suggest that CgATG16 contributes to the pathogenicity of *C. gloeosporioides* to the rubber tree by regulating the mycelium growth, melanin synthesis and the formation of invasion structure, and this process is related to autophagy mediated by TOR and cAMP signaling.

## 1. Introduction

Autophagy in eukaryotes is a highly conserved process to maintain homeostasis by degradation and recycling of damaged organelles or superfluous cellular components [1]. The process of autophagy is modulated by a series of autophagy-related (ATG) proteins [2]. Multiple studies have demonstrated that autophagy plays important roles in pathogenicity of various fungal pathogens [1]. At present, over 40 ATGs have been identified in yeast and their homologs are well conserved in the sequenced plant pathogenic fungi [3]. These ATGs are widely involved in the regulation of pathogenicity in plant pathogenic fungi [4,5,6].

ATG16, a subunit of the ATG12-ATG5-ATG16 complex which functions in a manner analogous to an E3-like enzyme, is required for ATG8 lipidation, a process of ATG8 conjugation to phosphatidylethanolamine in the autophagosomal membrane, which is an essential step for the phagophore membrane expansion and the autophagosome formation [7,8]. Studies in yeast have shown that ATG16 is required for autophagy and functions structurally as a homodimer mediated by a coiled-coil domain (CCD) which is also responsible for the combination of ATG16 and ATG12 [9,10]. In yeast, ATG16 forms homodimers via its coiled-coil domain (CCD) and interacts with the ATG12-ATG5 conjugate through its N-terminal ATG5-binding motif to assemble the functional complex [11]. In human pathogenic *Acanthamoeba castellanii*, AcATG16 was involved in autophagosome formation of mitochondrial autophagy and played an essential role in the encystation [12]. In the insect-pathogenic fungus *Beauveria bassiana*, deletion of *BbATG16* resulted in defects in asexual development, tolerance to oxidative stress, virulence and autophagosome formation [13].

Current research on the role of ATG16 in plant pathogenic fungi is limited, with only one reported study in *Magnaporthe oryzae*. *M. oryzae* ATG16 (MoATG16) physically interacted with Clp1, a PHD domain-containing protein, and colocalized at preautophagosomal structures (PASs) and autophagosomes, and the loss of Clp1 displayed defects in fungal morphology and development, glycogen metabolism, plant infection and virulence [14]. However, the precise molecular function of MoATG16 in autophagy regulation and the mechanistic basis of MoATG16-Clp1 remain unclear.

Natural rubber, as an irreplaceable important strategic resource and industrial raw material, plays an important role in the transportation and military industries. As the main source of natural rubber, rubber tree (*Hevea brasiliensis*) is an economically vital tropical tree species in numerous tropical countries, whose productivity directly underpins the sustainability of the global natural rubber industry [15]. *Colletotrichum gloeosporioides* can cause rubber tree anthracnose and result in serious loss of natural rubber production [16]. In this study, *C. gloeosporioides ATG16* (*CgATG16*) was amplified and its functional roles in the development and virulence by generating deletion mutant (Δ*CgATG16*) was studied. This study enriched our understanding of the roles of ATG16 in plant pathogenic fungi.

## 2. Materials and Methods

### 2.1. Fungal Strains and Culture Conditions

The wild-type (WT) strain of *Colletotrichum gloeosporioides* used in this manuscript was isolated in 2015 from anthracnose-infected leaves of *Hevea brasiliensis* at the Xiqing plantation in Danzhou City, Hainan Province. The fungal strains used in this study were cultured on potato dextrose agar (PDA: 200.0 g/L potato extract, 20.0 g/L dextrose, 20.0 g/L agar; pH 6.0) at 28 °C under dark conditions [17].

### 2.2. Bioinformatics Analysis

The sequence of *CgATG16* was derived from the transcriptome database of *C. gloeosporioides* (transcript ID: PV866801). The ORF sequence of ATG16 was obtained from a transcriptome database of *C. gloeosporioides*. Using this sequence as the template, specific amplification primers for *CgATG16* were designed by using Primer Premier 5.0 software. The sequence of *CgATG16* was subsequently verified by RT-PCR amplification and sequencing. All primers synthesized by Beijing Tsingke Biotechnology Co., Ltd. (Beijing, China) are listed in Supplementary Table S1. The amino acid sequence of CgATG16 was deduced by DNAMAN (version 7,0,2,176) software. Signal peptides and transmembrane domains were predicted using the online tools NovoPro SignalP 5.0 (https://novopro.cn/tools/signalp.html (accessed on 10 April 2024)) and DeepTMHMM 1.0.39 (https://dtu.biolib.com/DeepTMHMM (accessed on 10 April 2024)), respectively. Conserved domains were predicted using SMART 7 (http://smart.embl-heidelberg.de/ (accessed on 13 September 2024)). Protein secondary structures were predicted using JPRED 4 (https://www.compbio.dundee.ac.uk/jpred/ (accessed on 15 December 2024)). Sequence alignment of the N-terminal (ATG5-interacting motif) regions of different ATG16 proteins was carried out using the Multiple Sequence Alignment program at the NCBI COBALT tool (https://www.ncbi.nlm.nih.gov/tools/cobalt/ (accessed on 12 April 2024)). WebLogo (version 2.8.2) was used for making logos of N-terminal ATG5-interacting motif [18].

### 2.3. Quantitative RT-PCR Analysis

To explore the expression pattern of *CgATG16*, samples were prepared from *C. gloeosporioides* mycelia, conidia, germinated conidia, appressoria, and rubber tree leaves inoculated with the fungus, following previously described protocols. For mycelial RNA extraction, conidial suspensions were inoculated into complete medium (CM) to an initial concentration of 1 × 10^3^ conidia·mL^−1^, and incubated at 28 °C with shaking at 120 rpm for 2 days. Mycelia were then harvested for subsequent RNA isolation. For germinated conidium and appressorium samples, conidial suspensions (1 × 10^5^ conidia·mL^−1^) were inoculated onto plastic plates and incubated at 28 °C for 4 h (for germinated conidia) and 24 h (for appressoria), respectively. The germinated conidia and appressoria were collected using a cell scraper. For RNA extraction during the fungal infection of rubber tree leaves, conidial suspensions were sprayed onto rubber tree leaves. Inoculated leaves were harvested at 0, 1, 2, and 3 days post-inoculation (dpi), respectively, and immediately frozen at −80 °C for total RNA extraction. Fungal total RNA was extracted using Trizol Reagent (Invitrogen, Waltham, MA, USA), following the method described previously. For plant total RNA extraction, the Polysaccharide- and Polyphenol-Rich Plant Total RNA Extraction Kit (TIANGEN Biotech, DP441) was used according to the manufacturer’s instructions. Reverse transcription was performed using SPARK script II All-in-one RT SuperMix for qPCR (With gDNA Eraser) (sparkjade, AG0305-B, Shandong, China). Quantitative real-time PCR (qRT-PCR) analysis was conducted on a Quant Studio™ 6 Flex Real-Time PCR System (Applied Biosystems, Foster City, CA, USA). The designed primers were validated for specificity using NCBI Primer Blast (https://www.ncbi.nlm.nih.gov/tools/primer-blast/index.cgi?LINK_LOC=BlastHome (accessed on 15 July 2024)). The β-tubulin-1 gene (Cgβ-tub1) was used as the internal reference gene for normalizing gene expression levels in *C. gloeosporioides*. The relative expression levels of the target genes were calculated using the 2^−ΔΔCt^ method, with four technical replicates for each sample [17].

### 2.4. Construction of CgATG16 Knockout and Complementary Strains

The *CgAtg16* gene knockout strains were constructed using the split-marker strategy based on the principle of homologous recombination [19]. Briefly, the coding region of the CgATG16 on the genome was determined based on the ORF sequence of *CgATG16*. Then, based on the genomic sequence of *C. gloeosporioides*, the upstream of the coding region of the *CgATG16* (5′-flanking regions, 624 bp) and the downstream of the coding region of the *CgATG16* (3′-flanking regions, 761 bp) were, respectively, amplified from the genomic DNA of *C. gloeosporioides*. Meanwhile, the acetolactate synthase gene (SUR) cassette, which confers resistance to chlorimuron ethyl (a sulfonylurea herbicide) to the strain, was cloned from the vector pCB1532. Subsequently, primer pairs CgAtg16-5F/SURspl-R and SURspl-F/CgAtg16-3R were used, respectively, to amplify the fusion fragments of “flanking region-truncated SUR resistance gene. These fragments served as materials for subsequent protoplast transformation [20].

Protoplast preparation and transformation were performed as described in our laboratory-established protocol [21]. Conidia were inoculated into 200 mL of potato broth to an initial concentration of 10^5^ conidia·mL^−1^, and the culture was incubated at 28 °C with shaking at 150 rpm for 24 h. Mycelia were then harvested by filtration through a nylon membrane, washed twice with 1 M sorbitol, and transferred to 1 M sorbitol supplemented with 10 mg·mL^−1^ lysing enzyme (Sigma-Aldrich (Oakville, ON, Canada)). The mycelial suspension was incubated at 28 °C with shaking at 100 rpm for 3 h to induce cell wall degradation. Protoplasts were collected via filtration through a nylon membrane, followed by centrifugation at 2000 rpm and 4 °C. After two additional washes, the protoplasts were resuspended in STC buffer (1 M sorbitol, 50 mM Tris-HCl, 10 mM CaCl_2_, pH 7.4) to a final concentration of 10^8^ CFU·mL^−1^. For the transformation process, 100 mg of the fusion fragment was mixed with 200 μL of protoplasts, and the mixture was incubated on ice for 20 min. Next, 1 mL of 40% polyethylene glycol (PEG, dissolved in STC buffer) was added, and the mixture was further incubated at 28 °C for 20 min. An amount of 5 mL of liquid regeneration medium (composed of 1 g·L^−1^ yeast extract, 1 g·L^−1^ casein, and 6 M sucrose) was added to the mixture, which was then incubated at 28 °C with shaking at 100 rpm for 4 h. The regenerated protoplasts were mixed with regeneration medium (preheated to 50 °C) containing 1% agar, gently homogenized, and spread onto Petri dishes. Once the agar solidified, an equal volume of regeneration medium supplemented with 1% agar and 100 μg·mL^−1^ chlorimuron ethyl was overlaid to select transformants.

Two independent PCR reactions using primers CgATG16-d5F/R and CgATG16-d3F/R were conducted to verify the correct integration of the resistant transformants. Positive transformants were further verified by (1) single-conidium purification (to eliminate heterokaryons); and (2) sequencing of homologous recombination junctions (performed by Tsingke Biotechnology (Beijing, China)). To generate the complementary strain, the sequences of *CgATG16* with its promotor were ligated into the complementary vector pGPDA with HPT (hygromycin phosphotransferase gene) cassette. Protoplast preparation and transformation of *CgATG16* knockout mutant were performed as above. The positive transformants of complementary strains were further confirmed through the PCR diagnosis of *CgATG16* ORF.

### 2.5. Pathogenicity Assay

Conidia of *C. gloeosporioides* were suspended in a solution of 0.5% Malt Extract Broth (Difco, Franklin Lakes, NJ, USA) to a final concentration of 2 × 10^5^ conidia/mL. Using 0.5% Malt Extract Broth as negative control, 5 µL of conidial suspensions were inoculated onto the wounded detached “light green” leaves of rubber tree variety 73-3-97. The inoculated leaves were kept in a moist chamber at 28 °C under natural illumination and the disease symptoms were scored at 4 days post-inoculation.

A conidial suspension with a concentration of 2 × 10^5^ conidia/mL was used, and 5 μL droplets of this suspension were inoculated onto the pre-wounded sites of detached “light green” leaves of the rubber tree cultivar 7-33-97, which were maintained in a greenhouse. The disease symptoms were scored at 4 days post inoculation. Each experiment contained three replicates of at least 10 leaves.

### 2.6. Fungal Growth, Conidiation and Germination Assay

For fungal growth assay, 5 mm diameter hyphae agar disks were taken and inoculated into PDA medium for 5 days, and the colony morphology were observed and the colony diameters were recorded.

For conidiation assay, conidia of indicated strains were harvested from the strains growing on PDA medium for 7 days and inoculated into 50 mL liquid complete medium (CM) (1% Glucose, 0.1% Trace element solution, 0.1% Vitamin solution, 5% Nitrate solution, 0.2% Peptone, 0.1%Yeast extract, and 0.1% Acid—hydrolyzed casein per liter) to the final conidia concentration of 10^4^/mL. The conidia numbers were counted under a microscope after incubation at 28 °C with shaking (120 rpm) for 3 days.

For conidia germination assay, spores were resuspended in 1 mL of 2% YCS (2% sucrose, 0.1% yeast extract, 0.1% ac-id-hydrolyzed casein), centrifuged at 5000 r/min for 30 s, and this centrifugation–washing step was repeated twice. The spore pellet was resuspended in 1 mL of 2% YCS, and the spore concentration was adjusted to 5 × 10^5^ spores/mL. A 20 μL aliquot of the spore suspension was inoculated onto clean Petri dishes, which were then moisturized and incubated in a 28 °C incubator. Samples were prepared at 2 h and 4 h for microscopic observation; images were captured, and statistical analysis of germination rates was performed.

The experiments were repeated three times, with four replicates for each sample and ten microscope fields surveyed for every replicate.

### 2.7. Assays for Fungal Biomass and Melanin Content

A piece of cellophane was cut to fit the size of a Petri dish, weighed, and then sterilized. The sterilized cellophane was moistened with sterile water and carefully placed onto fresh PDA medium using sterile tweezers. Freshly cultured test strains (wild-type, mutant, and corresponding complemented strain) with consistent growth status were punched into agar plugs of uniform size using a sterile cork borer. Each agar plug was inoculated at the center of the cellophane, and the Petri dishes were labeled accordingly. All cultures were sealed in plastic bags and incubated at 28 °C for 5 days. After measuring the colony diameter using the cross method, the cellophane with the mycelium was collected, dried, and weighed. All data were analyzed using one-way analysis of variance (ANOVA) followed by Tukey’s honestly significant difference (HSD) test to determine significant differences between groups. Statistical significance was defined as a *p*-value < 0.05. The experiment was independently repeated three times.

For melanin content determination, the procedure was performed following the instructions of the GENMED Fungal/Yeast Melanin Colorimetric Quantification Kit (GMS50365.3). Briefly, the concentration of fresh spore suspensions of the strains was adjusted to 1 × 10^4^ spores/mL, and conidiospores were inoculated into CM medium. The cultures were incubated at 28 °C with shaking at 160 rpm for 3 days, after which the mycelia were filtered, collected, and dried. The dried mycelia were rapidly ground into a fine powder in liquid nitrogen. For each mycelial sample, three aliquots of 0.05 g each were weighed out for subsequent melanin extraction. Melanin was extracted according to the kit instructions, and its content was quantified using a microplate reader at a wavelength of 360 nm. The absorbance values were recorded, and the melanin content of each sample was calculated using a standard curve (the standard curve was generated based on the measurements of the standard supplied with the kit) (unit: μg/mg DW). Three biological replicates were performed for this assay.

### 2.8. Appressorium Development and Penetration Ability Assay

For appressorium development assay, 20 μL drops of conidial suspensions were placed on a hydrophobic plastic Petri dish and incubated at 28 °C. The conidial morphology was observed at 0, 2, 4, and 8 h post-incubation, and the percentages of appressorium formation were determined under a microscope at 8 h post-incubation. Moreover, in order to identify the potential signaling pathway by which CgATG16 functions, the following treatments at the respective final concentrations were added to the conidial suspensions and analyzed at 12 h:200 nM rapamycin (Rap; Beyotime Biotechnology, Shanghai, China) and 10 mM 8-bromoadenosine 3′,5′-cyclic monophosphate sodium salt (8-Br-cAMP; Sparkjade, Jinan, China).

The invasive growth assay was performed on onion epidermis which was sprayed with 2.5 × 10^5^/mL of conidia and placed on agar medium plate at 28 °C for 12 h, and the invasion was observed under a microscope. The experiments were repeated three times, and at least 100 conidia were detected per replicate. The conidia were randomly selected from different microscopic fields of view.

### 2.9. Analysis of Nitrogen Starvation Induced Autophagy

Fresh spore suspensions were prepared using liquid CM medium, and 20 mL of spore suspension with a concentration of 5 × 10^5^ spores/mL was obtained. The spore suspension was cultured at 28 °C with shaking at 120 rpm for 16–24 h to harvest mycelia.

The cultured mycelia were aspirated and evenly distributed into several 2 mL centrifuge tubes. For each tube, 1.5 mL of sterile ddH_2_O was added, followed by gentle shaking to mix thoroughly. The mixture was centrifuged at 4000 rpm for 1 min; the supernatant was carefully discarded, and residual liquid at the bottom was removed. This centrifugation–washing step was repeated twice to eliminate nutrients from the CM medium. Subsequently, the mycelia were treated with MM-N (MM-N: 0.05% Potassium chloride, 0.1% Potassium dihydrogen phosphate, 0.05% Magnesium sulfate heptahydrate, 0.001% Ferrous sulfate heptahydrate, 3% sucrose, and 0.02% Trace element solution per liter) medium supplemented with PMSF solution (final concentration: 4 mmol/L) to induce autophagy under nitrogen starvation conditions, and cultured at 28 °C with shaking at 120 rpm for 2 h and 5 h, respectively. Among them, PMSF can inhibit vacuolar degradation, which facilitates the observation of the presence of autophagosomes within vacuoles [22]. Observations were performed under a fluorescence microscope, followed by statistical analysis. The experiment was independently repeated three times, with no fewer than ten fields of view observed per repetition.

### 2.10. Protein Extraction and Western Blot Analysis

Spore suspensions of the strains were cultured in liquid CM medium at 28 °C with shaking at 120 rpm for 24 h. The mycelia were washed with ddH_2_O and then transferred to MM-N medium, followed by incubation at 28 °C with shaking at 120 rpm for 2 h and 5 h, respectively. Mycelia were collected, ground into powder in liquid nitrogen, and resuspended in 1 mL of lysis buffer (Lysis Buffer: 50 mM Tris-HCl (pH 8.0), 150 mM NaCl, 1 mM EDTA, 1% Triton-X-100, 1 mM PMSF, and 1× protease inhibitor cocktail per liter) containing a protease inhibitor cocktail. The mixture was vortexed to ensure thorough mixing, and the lysate was centrifuged at 12,000× *g* for 20 min at 4 °C. The supernatant containing proteins was collected. The protein concentration was determined and quantified using the Bradford Protein Assay Kit (detergent-compatible) (Beyotime, P0006C, Shanghai, China). Subsequently, proteins were analyzed by 12% SDS-PAGE and then subjected to Western blot using a 1:10,000 dilution of anti-GFP primary antibody (ABclonal, AE012, Wuhan, China), and the anti-GAPDH primary antibody (ProteinTech Group, 60004-1-Ig, Wuhan, China) was used as an internal reference control. A Goat anti-Mouse IgG (H + L) (Thermo, 31160, Waltham, MA, USA) was used as the secondary antibody with a dilution of 1:20,000. Finally, protein signals were detected using the BeyoECL Plus chemiluminescence kit (Beyotime, P0018S, Shanghai, China) and analyzed with ImageJ (version 1.53e) software.

The phosphorylation level of p70-S6 kinase 1 (S6K1), a functional orthologue of yeast Sch9 and a TOR substrate, was used to indirectly reflect the activity of TOR [23]. To detect S6K/Sch9 phospho-status, WT, Δ*CgATG16* and Res-Δ*CgATG16* were grown in CM, as above, and the mycelia were transferred to fresh CM with and without 200 nM Rap for 8 h. Mycelia harvested from the second growth regime were washed with distilled water three times and finely ground in liquid nitrogen. Equal amounts of mycelia powder were used for total protein extraction in a freshly prepared cell lysis buffer (60 mM Tris-HCl, pH 6.8, 2% SDS, 10% (*w*/*v*) glycerol, 5% β-mercaptoethanol) supplemented with protease inhibitors (5 mM EDTA, 1 mM PMSF, 1× cocktail) and phosphatase inhibitors (5 mM Na_3_VO_4_), followed by denaturation at 95 °C for 3 min. The cell lysates were cleared by centrifugation at 16,000× *g* for 15 min at 4 °C, and equal volumes of total proteins in lysates were resolved by 10% SDS-PAGE and then transferred to a PVDF membrane. The phosphorylation status of S6K1/Sch9 was monitored using an anti-phospho-p70 S6 kinase antibody (Beyotime Biotechnology, Shanghai, China) diluted 1:5000 in Western Primary Antibody Dilution Buffer (Sparkjade, ED0013), and the signals were normalized to those of p70 S6 kinase and GAPDH, which served as internal references. Protein signals were detected using the BeyoECL Plus chemiluminescence kit (Beyotime, P0018S, Shanghai, China) and analyzed with ImageJ software.

### 2.11. cAMP Content Assay

After 24 h of massive induction of appressorium formation on hydrophobic surfaces, the appressoria were collected into 1.5 mL centrifuge tubes using a cell scraper, followed by weighing. Dilute HCl (0.1 mol/L) was then added at a ratio of 10% (*w*/*v*). After dissolution by vortexing, the mixture was centrifuged at 1000 rpm for 10 min at room temperature, and the supernatant was collected. The cyclic adenosine monophosphate (cAMP) content was determined according to the instructions of the Fungus cAMP ELISA KIT (GOY-L0141, Shanghai Guyan, Shanghai, China).

### 2.12. Statistical Analysis

Statistical significance analyses were performed using SPSS Statistics version 21.0. Data with a single variable were analyzed by one-way analysis of variance, and mean separations were performed by Duncan’s multiple range test. Differences at *p* < 0.05 were considered significant.

## 3. Results

### 3.1. Cloning and Analysis of CgATG16 in C. gloeosporioides

**Figure 1:**
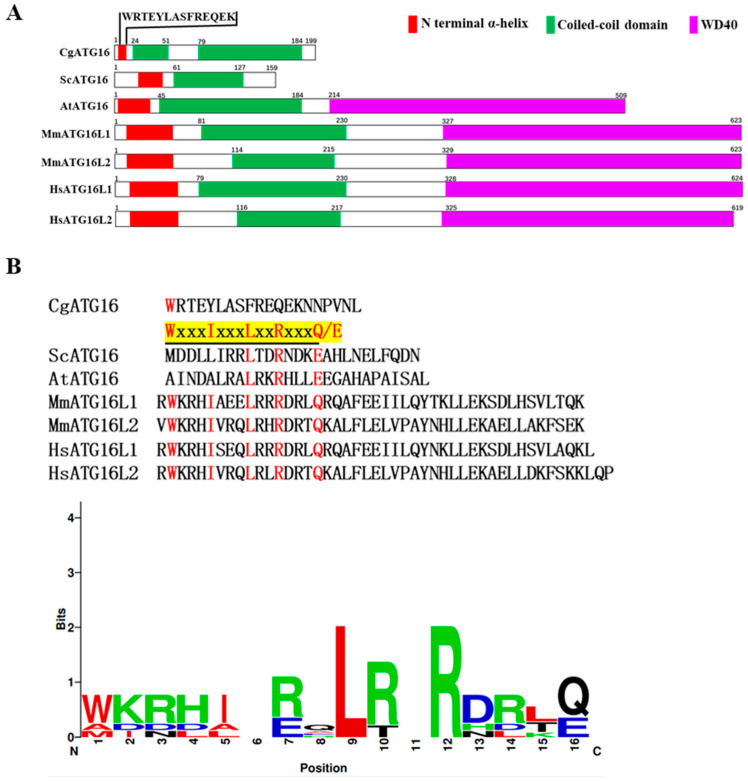
Protein structure analysis of CgATG16 and homologs from different species. (**A**) Domain structures of CgATG16, *Saccharomyces cerevisiae* ATG16 (NP_013882.1), *Arabidopsis thaliana* (NP_199834.2), *Mus musculus* ATG16L1 (NP_001192320.1) and ATG16L2 (NP_001104581.1), *Homo sapiens* ATG16L1 (NP_001350671.1) and ATG16L2 (NP_203746.1) were predicted using SMART 7 (http://smart.embl-heidelberg.de/ (accessed on 13 September 2024)). N-terminal α-helix (ATG5-interacting motif) were predicted using JPRED 4 (https://www.compbio.dundee.ac.uk/jpred/ (accessed on 15 December 2024)). (**B**) Sequence alignment of N-terminal α-helix regions of different ATG16 proteins using the Multiple Sequence Alignment program at the NCBI (https://blast.ncbi.nlm.nih.gov/Blast.cgi (accessed on 01 July 2024)). The yellow shading denoted on the top indicates the consensus sequence of ATG5-interacting motif (x represents any residue) and red letters highlight conserved key residues of ATG5-interacting motif [24,25]. The sequence logos were made using WebLogo 2.8.2 [18].

Homology search was conducted using the *ATG16* gene sequence of *Saccharomyces cerevisiae* in the transcriptome database of *C. gloeosporioides*, and the sequence information (ORF) of transcript (PV866801) was obtained, which was named *CgATG16*. The open reading frame of *CgATG16* was amplified by RT-PCR. The sequencing results showed that the ORF of *CgATG16* contained 600 bp encoding a polypeptide of 200 amino acids with molecular weight of 22.6 kDa, theoretical pI of 6.08, a conserved autophagy-related protein 16 domain (Figure S1). Signal peptide and transmembrane domain predictions using NovoPro SignalP 5.0 (https://novopro.cn/tools/signalp.html (accessed on 10 April 2024)) and DeepTMHMM 1.0.39 (https://dtu.biolib.com/DeepTMHMM (accessed on 10 April 2024)) indicated that CgATG16 is a cytoplasmic protein lacking targeting sequences. Existing studies have shown that ATG16 proteins of different species all possess an N-terminal α-helix (ATG5-interacting motif) and followed by the coiled-coil domain (CCD). In addition, a C-terminal WD40 repeat was present in most of the higher eukaryotes ATG16 proteins [24,25]. Therefore, we analyzed the secondary structure of CgATG16 using SMART 7 (http://smart.embl-heidelberg.de/ (accessed on 13 September 2024)) and JPRED 4 (https://www.compbio.dundee.ac.uk/jpred/ (accessed on 15 December 2024)) program. Our analysis showed that CgATG16 contained an N-terminal α-helix (4-17 aa) and two CCD (24-51 aa, 79-184 aa), but lacked WD40 repeats, which was most similar to that of *Saccharomyces cerevisiae* ATG16 (Figure 1A). Among them, N-terminal α-helix of CgATG16 was composed of 13 amino acid residues “WRTEYLASFREQEK” (Figure 1B). The sequence alignment showed that the N-terminal α-helix sequence of CgATG16 had an extremely low homology with ATG5-binding motifs from different species, and lacked the consensus sequence of ATG5-interacting motif “WxxxIxxxLxxRxxxQ/E” (Figure 1B).

### 3.2. CgATG16 Was Induced by Appressorium Development, Infection, and Nutritional Deficiency

**Figure 2:**
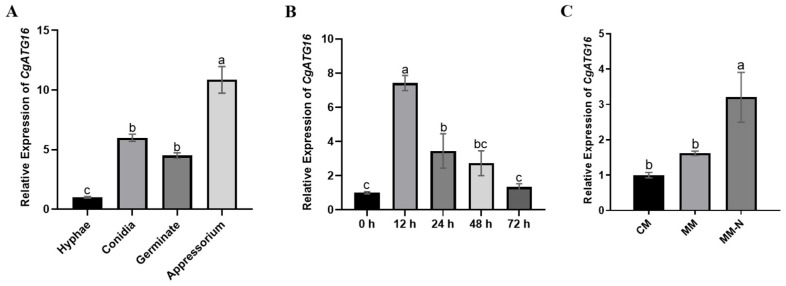
Analysis of CgATG16 expression patterns. (**A**) Analysis of *CgATG16* gene expression levels in *C. gloeosporioides* at different growth and developmental stages. (**B**) Analysis of *CgATG16* gene expression levels in *C. gloeosporioides* at different time points during the infection of rubber tree leaves. (**C**) Analysis of *CgATG16* gene expression levels in *C. gloeosporioides* after 24 h induction in different media. Data are presented as the mean ± standard deviation (SD) from three independent experiments. Duncan’s multiple range test was used to analyze the statistical significance of differences between groups for all column charts, where columns marked with different letters indicate statistically significant differences (*p* < 0.05).

In order to explore the possible role of *CgATG16* in the autophagy, growth and development, and infection process of *C. gloeosporioides*, the expression levels of *CgATG16* were examined in mycelia, conidia, appressorium, nutritional deficiency and during infection. The results showed that the expression levels of *CgATG16* in conidium, germination, and appressorium were significantly increased compared to that in mycelia, and the expression level in appressorium was significantly higher than those in germination and conidium (Figure 2A). During infection of *C. gloeosporioides* toward rubber tree leaves, the expression level of *CgATG16* was induced more than 7 times at 12 h post-inoculation and then gradually decreased to the original level at 72 h post-inoculation (Figure 2B). Additionally, the expression of *CgATG16* was significantly induced by nitrogen deficiency (Figure 2C).

### 3.3. CgATG16 Contributed to Pathogenicity

**Figure 3:**
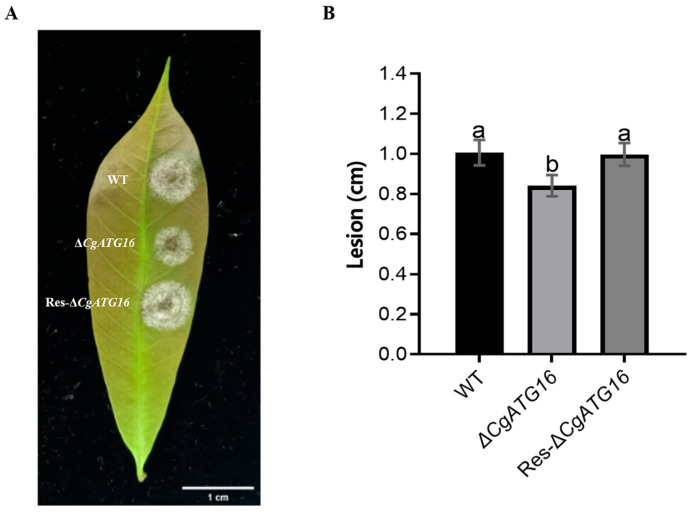
Pathogenicity analysis of Δ*CgATG16* on rubber tree leaves. (**A**) Lesion formation on rubber tree leaves 3 days after inoculation with the wild-type (WT) strain, Δ*CgATG16* mutant and Res-ΔCgATG16 strain. Scale bar = 1 cm. (**B**) Statistical analysis of lesion diameters on the leaves. Data are presented as the mean ± standard deviation (SD) from three independent experiments. Duncan’s multiple range test was used to analyze the statistical significance of differences between groups for all column charts, where columns marked with different letters indicate statistically significant differences (*p* < 0.05).

To investigate the functions of *CgATG16* in *C. gloeosporioides*, the *CgATG16* gene deletion mutant (Δ*CgATG16*) and complementation mutants (Res-Δ*CgATG16*) were generated through homologous recombination and gene insertion strategy (Figure S2A,C). Single-conidium purification and junction sequencing were performed to ensure the purity and integrity of the mutants, while PCR analysis was conducted to verify the successful generation of both Δ*CgATG16* and Res-Δ*CgATG16* mutants (Figure S2B,D). The detached leaf inoculation assay showed that wild-type (WT), Δ*CgATG16* and Res-Δ*CgATG16* caused typical necrotic lesions at 4 days post-inoculation (Figure 3A), while the mock inoculation with 0.5% Malt Extract Broth showed no typical necrotic lesions (Figure S3). Statistical analysis showed that the size of necrotic lesions induced by Δ*CgATG16* was significantly smaller than the size of those induced by WT, and Res-Δ*CgATG16* restored the virulence of Δ*CgATG16* (Figure 3B). These data indicated that *CgATG16* contributed to the pathogenicity of *C. gloeosporioides* to the rubber tree.

### 3.4. CgATG16 Contributed to Growth, Mycelial Biomass and Melanin Production

**Figure 4:**
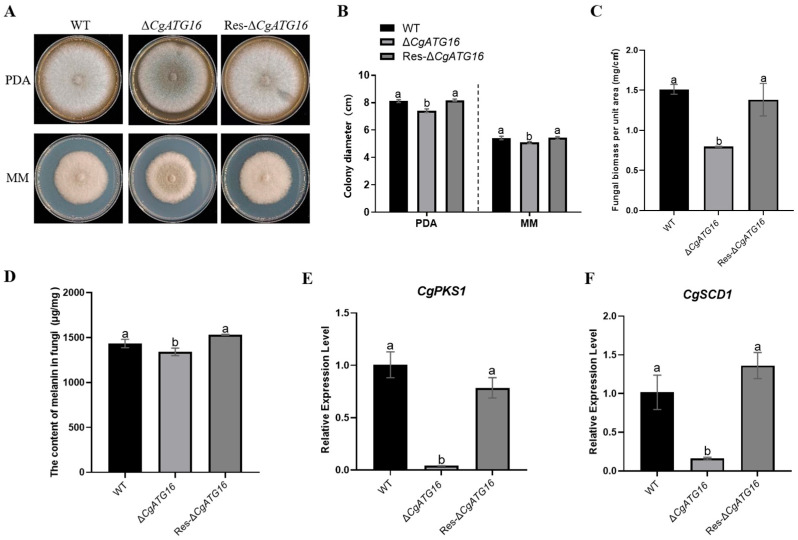
Analysis of colony growth and melanin content in Δ*CgATG16*. (**A**) Colony morphology on PDA and MM media. (**B**) Statistical analysis of colony diameters on PDA and MM media. (**C**) Biomass analysis of different strains. (**D**) Melanin content analysis of in different strains. (**E**) Relative expression levels of *CgPKS1* in different strains. (**F**) Relative expression levels of *CgSCD1* in different strains. Data are presented as the mean ± standard deviation (SD) from three independent experiments. Duncan’s multiple range test was used to analyze the statistical significance of differences between groups for all column charts, where columns marked with different letters indicate statistically significant differences (*p* < 0.05).

To explore whether CgATG16 affects the colony growth of *C. gloeosporioides*, the growth of WT, Δ*CgATG16* and Res-Δ*CgATG16* were analyzed on PDA and MM media, respectively. Overall, the colony growth rate of WT, Δ*CgATG16* and Res-Δ*CgATG16* on MM media was visibly lower than that on PDA media (Figure 4A). Whether on PDA media or on MM media, although statistical analysis revealed that the colony diameter of Δ*CgATG16* was always significantly smaller than that of WT, while the colony diameter of Res-Δ*CgATG16* was comparable to that of WT, the absolute difference was minor (Figure 4B).Additionally, we also noticed that the color and thickness of mycelium of Δ*CgATG16* colony were different from WT and Res-Δ*CgATG16* on both PDA media and MM media (Figure 4A). Further analysis revealed that the mycelium biomass per unit area of Δ*CgATG16* was significantly lower than that of WT and Res-Δ*CgATG16* (Figure 4C), and the content of melanin per unit mass was significantly lower than that of WT and Res-Δ*CgATG16* (Figure 4D). Polyketide synthase gene *CgPKS1* and scytalone dehydratase gene *CgSCD1* were essential for melanin biosynthesis in *C. gloeosporioides* [26,27]. The expression level of *CgPKS1* and *CgSCD1* in Δ*CgATG16* were reduced to approximately one-twentieth and one-eighth compared with that in WT and Res-Δ*CgATG16*, respectively (Figure 4E,F). These results indicated that CgATG16 was involved in colony growth rate, biomass and melanin production of *C. gloeosporioides*, and the involvement of *CgATG16* in melanin production by regulating *CgPks1* and *CgSCD1*.

### 3.5. CgATG16 Contributed to Sporulation, Conidia Germination, Appressorium Formation and Primary Invasion

**Figure 5:**
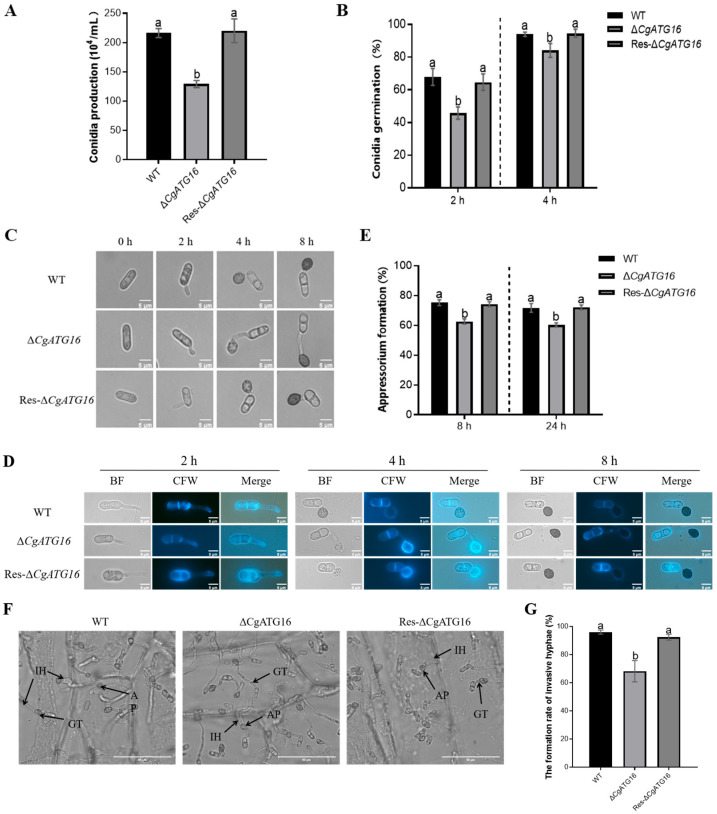
Analysis of sporulation, conidia germination, appressorium development and primary infection of Δ*CgATG16.* (**A**) Conidial yield analysis of different strains. (**B**) Statistical analysis of spore germination rates of different strains at 2 h and 4 h. (**C**) Microscopic observation of the appressorium development (scale bar = 5 μm). (**D**) CFW staining of WT, Δ*CgATG16*, and Res-Δ*CgATG16* at different developmental time points (2 h, 4 h, and 8 h). Scale Bar = 5 µm. (**E**) Statistical analysis of appressorium formation rates. (**F**) Invasion observation in onion epidermal cells at 12 h post-inoculation. (AP, IH, GT indicate the appressorium, invasive hyphae and germ tube, separately. Scale Bar = 50 µm.) (**G**) Statistical analysis of invasive hyphae formation in WT, Δ*CgATG16* and Res-Δ*CgATG16* at 12 h post-incubation. Data are presented as the mean ± standard deviation (SD) from three independent experiments. Duncan’s multiple range test was used to analyze the statistical significance of differences between groups for all column charts, where columns marked with different letters indicate statistically significant differences (*p* < 0.05).

In order to explore whether *CgATG16* is involved in conidia production, conidia germination and appressorium development of *C. gloeosporioides*, the conidia yield, conidia germination rates and appressorium formation and development of WT, Δ*CgATG16* and Res-Δ*CgATG16* were analyzed, respectively. The results showed that the conidia yield of Δ*CgATG16* was significantly lower than that of WT, and the conidia yield of Res-Δ*CgATG16* recovered to the level of WT (Figure 5A). The statistical results of spore germination rates showed that the spore germination rate of Δ*CgATG16* was significantly lower than that of WT at both 2 h and 4 h (Figure 5B). The developmental process from conidia germination to the appressorium formation among WT, Δ*CgATG16* and Res-Δ*CgATG16* was systematically compared in vitro. Microscopic examination revealed that, apart from the fact that the germ tubes of Δ*CgATG16* were significantly longer than those of WT, all the conidia of WT, Δ*CgATG16* and Res-Δ*CgATG16* normally germinated to form germ tubes in 2 h post incubation and then formed normal appressorium at the tip of germ tubes in 8 h post incubation (Figure 5C). To determine whether these changes in germ tube length were due to cell size or cell numbers, calcofluor white (CFW) staining was performed. The results showed that there was no significant difference in the septum number of germ tubes between Δ*CgATG16* and WT strains (Figure 5D). The appressorium formation rate of Δ*CgATG16* was significantly lower than that of WT and Res-Δ*CgATG16* (Figure 5E). In addition, the primary invasion of Δ*CgATG16* was tested on onion epidermis and observed under optical microscope. Compared to WT and Res-Δ*CgATG16* which could successfully complete infection and form normal primary hyphae in onion epidermis cells, no more than 70% of Δ*CgATG16* formed primary hyphae in onion epidermis cells (Figure 5F,G). These results demonstrated that the absence of *CgATG16* led to a significant reduction in the spore production, germination and appressorium formation rate, and invasive hyphae formation of *C. gloeosporioides*, as well as abnormal germ tube length, suggesting that CgATG16 was a key modulator of infection structure formation and early infection processes in *C. gloeosporioide*.

### 3.6. CgATG16 Was Essential for Autophagy Occurrence in C. gloeosporioides

**Figure 6:**
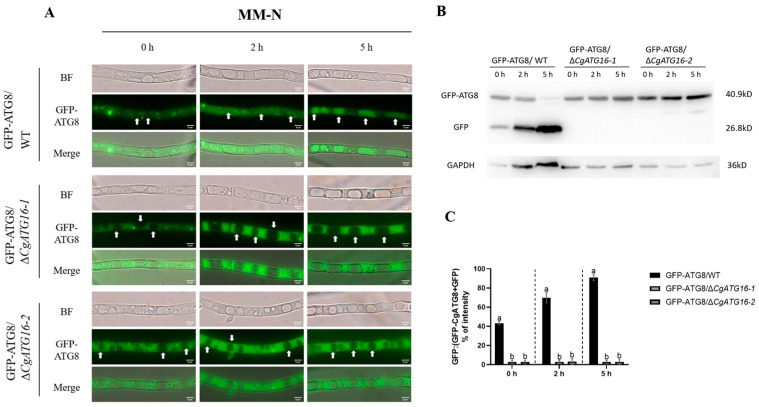
Analysis of nitrogen starvation-induced mycelial autophagy in Δ*CgATG16*. (**A**) Autophagy processes detected by observation of ATG8 subcellular localization. GFP-ATG8/WT and GFP-ATG8/Δ*CgATG16* strains was cultured in MM-N medium for 5 h, with arrows pointing to the vacuoles. Fluorescence changes were observed at 0 h, 2 h and 5 h, separately. Bars, 5 μm. (**B**) Immunoblot analysis conducted using anti-GFP methods after nitrogen deprivation treatment, with anti-GAPDH antibody used as a control. (**C**) The level of autophagy was estimated by calculating the amount of free GFP relative to the total amount of intact GFP-ATG8 plus free GFP. Data are presented as the mean ± standard deviation (SD) from three independent experiments. Duncan’s multiple range test was used to analyze the statistical significance of differences between groups for all column charts, where columns marked with different letters indicate statistically significant differences (*p* < 0.05).

Given that ATG8 is a universal marker of autophagy, GFP-ATG8 was introduced into both WT and Δ*CgATG16* strains, named as GFP-ATG8/WT and GFP-ATG8/Δ*CgATG16*, to explored the potential of CgATG16 in regulating autophagy of *C. gloeosporioides*. PCR detection was performed to verified GFP-ATG8/WT and GFP-ATG8/Δ*CgATG16* strains (Figure S4A–C). We subjected the mycelia of GFP-ATG8/WT and GFP-ATG8/Δ*CgATG16* to nitrogen starvation (MM-N). Under condition of nitrogen deficiency (0 h incubation in MM-N), we noticed a certain localization of GFP-ATG8 in the vacuoles of WT cells, but almost absent in GFP-ATG8/Δ*CgATG16* cells. Following a 5 h incubation in MM-N, GFP-ATG8 is mainly located in the vacuoles of WT cells, while there is no fluorescence distribution in the vacuoles of GFP-ATG8/Δ*CgATG16* cells (Figure 6A). This finding indicated the constitutive autophagy deficiency in Δ*CgATG16* mutants, independent of nitrogen availability. Additionally, we quantitatively estimated the autophagy level using Western blot analysis by calculating the free GFP relative to the total amount of intact GFP-CfATG8 and free GFP together. Upon treating the WT mycelia with nitrogen starvation medium (MM-N), the proportion of free GFP notably increased from 43% at 0 h to 90% at 5 h. Conversely, in Δ*CgATG16* strains, the ratio of free GFP was extremely low, accounting for only 3%, and exhibited no significant change with variations in induction time. (Figure 6B,C). Collectively, these results indicated that the deletion of *CgATG16* led to the absence of autophagy, suggesting that CgATG16 was essential for autophagy occurrence in *C. gloeosporioides*.

### 3.7. CgATG16 Was Involved in Conidia Autophagy During Appressorium Development

**Figure 7:**
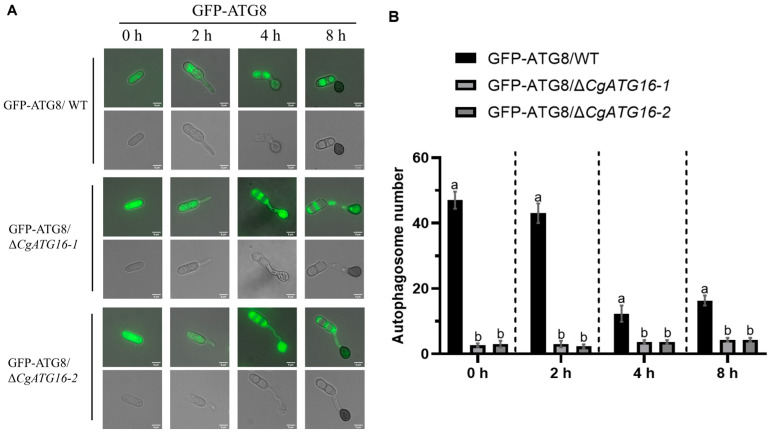
Analysis of autophagy in Δ*CgATG16* during appressorium formation and development. (**A**) Cellular localization of autophagosomes during appressorium formation. The conidia of different strains were inoculated onto a hydrophobic interface and observed at different time points using fluorescence microscopy. Bars, 5 μm. (**B**) Statistical analysis of the average number of autophagosomes at 0, 2, 4, and 8 h after germination. Data are presented as the mean ± standard deviation (SD) from three independent experiments. Duncan’s multiple range test was used to analyze the statistical significance of differences between groups for all column charts, where columns marked with different letters indicate statistically significant differences (*p* < 0.05).

To evaluate the relationship between the autophagy mediated by CgATG16 with appressorium development, we analyzed the formation of autophagosomes in both GFP-ATG8/WT and GFP-ATG8/Δ*CgATG16* strains. The results showed that autophagosomes labeled with GFP-ATG8 was typical and mainly accumulated in the conidia cells (0 h) and germinating conidia (2 h) of GFP-ATG8/WT strain, and there were almost none in GFP-ATG8/Δ*CgATG16*. With appressorium formation and maturation (4 h and 8 h), the autophagosomes labeled with GFP-ATG8 were mainly found in appressorium. There, although the overall number of autophagosomes was significantly lower than that in the conidia and germinating stages, the number of autophagosomes in both GFP-ATG8/WT was still significantly higher than that in GFP-ATG8/Δ*CgATG16* strain (Figure 7A,B). This data demonstrated that loss of CgATG16 led to autophagy defects during appressorium development, suggesting CgATG16 was required for autophagy in relation to appressorium development.

### 3.8. CgATG16 Contributed to Appressorium Development Through TOR and cAMP Signaling

**Figure 8:**
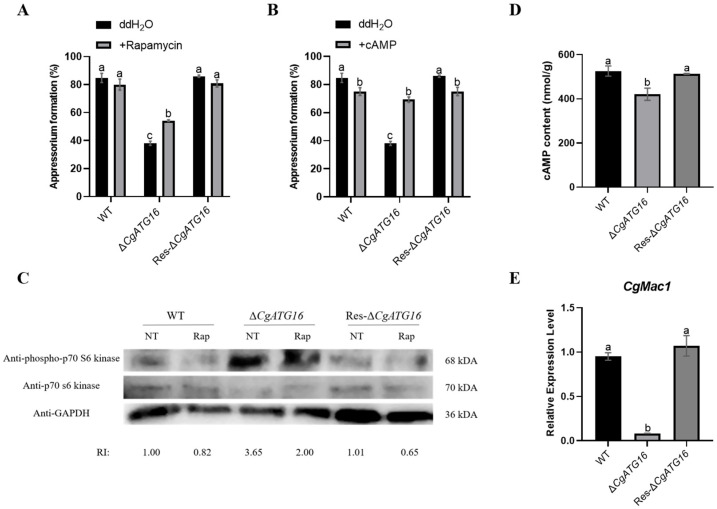
Effects of rapamycin and cAMP on the appressorium formation rates of WT, Δ*CgATG16* and Res-Δ*CgATG16*. (**A**) Appressorium formation rates of WT, Δ*CgATG16* and Res-Δ*CgATG16* at 12 h with 100 nM rapamycin. (**B**) Appressorium formation rates of WT, Δ*CgATG16* and Res-Δ*CgATG16* at 12 h with 10 mM cAMP. (**C**) Immunoblot showing the phosphorylation status of the direct TOR kinase target Sch9 in the indicated strains following treatment with 200 nM rapamycin (Rap) for 8 h. Strains were grown in liquid complete media (CM). NT = no treatment. RI = relative intensity, which is calculated by normalizing the Sch9 phosphorylation level detected with anti-phospho-p70 S6 kinase antibody to both the p70 S6 kinase level detected with anti-p70 S6 kinase antibody and the internal reference protein level detected with GAPDH antibody. (**D**) Analysis of cAMP content in appressoria of WT, Δ*CgATG16* and Res-Δ*CgATG16*. (**E**) Analysis of relative expression level of *CgMac1* in appressoria of WT, Δ*CgATG16* and Res-Δ*CgATG16* strains. Data are presented as the mean ± standard deviation (SD) from three independent experiments. Duncan’s multiple range test was used to analyze the statistical significance of differences between groups for all column charts, where columns marked with different letters indicate statistically significant differences (*p* < 0.05).

Given the close correlation between fungal autophagy mediated by the cAMP/PKA and TOR signaling and appressorium development, rapamycin (the specific TOR kinase inhibitor) and cAMP were used to identify the potential signaling pathway by which CgATG16 regulated the appressorium formation in *C. gloeosporioides*. The results showed that rapamycin could partially restore the appressorium formation rate of Δ*CgATG16* from 38.12% to 54.25% (Figure 8A), and cAMP basically restored the appressorium formation rate of Δ*CgATG16* (Figure 8B). As the TOR protein kinase negatively regulates autophagy and the appressorium formation rate of Δ*CgATG16* was partially restored by rapamycin, it was presumed that reduced TOR activity in Δ*CgATG16*. The p70-S6 kinase 1 (S6K1) is a functional orthologue of yeast Sch9 and a TOR substrate and the TOR activity can indirectly correspond with the phosphorylation level of S6K1. In order to verify this speculation, we determined the TOR activity in Δ*CgATG16* by detecting the phosphorylation level of S6K1 by Western blotting. By adding rapamycin, the phosphorylation level of S6K1 in the WT decreased, indicating that rapamycin could inhibit the TOR activity in *C. gloeosporioides*. However, the phosphorylation level of S6K1 in Δ*CgATG16* after adding rapamycin was much lower than that in WT (Figure 8C), indicating that the TOR activity of Δ*CgATG16* was reduced. In addition, the content of cAMP in Δ*CgATG16* was significantly lower than that in WT (Figure 8D). Consistently, the expression level of *CgMac1*, encoding an adenylate cyclase responsible for the conversion of ATP into cAMP, was significantly lower than that in WT (Figure 8E). These data suggested that CgATG16 regulated appressorium formation through modulating the activity of TOR and the synthesis of cAMP.

## 4. Discussion

Autophagosome formation is an essential step in the occurrence of autophagy [7,8]. ATG16, as one of the “core” ATG genes firstly identified in yeast in 1999, was present in all eukaryotic species and required for the formation of autophagosomes [11,28]. During canonical autophagy, ATG12-ATG5-ATG16 complex acts as an E3 ligase mediating Atg8 conjugation to phosphatidylethanolamine (PE) on membranes, thereby promoting the expansion of the autophagosome membrane [29]. The ATG16 proteins in eukaryotic species usually contained an N-terminal α-helix (ATG5-interacting motif) and had a CCD, as well as the WD40 domain that is unique to higher eukaryotic species, and was considered to be a scaffold protein that interacted with ATG12-ATG5 protein conjugates via its N-terminal ATG5-interacting motif and self-assembles through its coiled-coil domain (CCD) [24,30,31]. Our 199-amino-acid CgATG16 harbored an N-terminal α-helix and two CCDs, but lacked WD40 domain (Figure 1A), which was consistent with the structural characteristics of ATG16 proteins in lower eukaryotic species such as yeast. Studies in yeast had shown that yeast ATG16 contained a coiled-coil domain (CCD), which mediated both its own dimerization and interaction with ATG21 [31,32]. Recent studies had further confirmed that the dimerization and peripheral membrane-binding activity of yeast ATG16 depended on two distinct protein regions of CCD [9]. We noticed that CgATG16 contained two CCDs (Figure 1A); however, it was still unknown whether these two CCDs are related to dimerization and membrane-binding activity. By crystal structure assay, it was found that the 33 residues containing conserved sequence W-x-x-x-I-x-x-x-L-x-x-R-x-x-x-Q/E at N-terminal of human ATG16 are sufficient to bind to ATG5, which also was further confirmed by biochemical and cell biological analyses. In yeast, although only a partially conserved sequence “XXXXXXXX- L-x-x-R-x-x-x-Q/E” exists, ATG16 still interacts with ATG5 [24,25]. In this study, N-terminal helix of CgATG16 contained only 13 amino acid residues (WRTEYLASFREQEK), which has an extremely low homology with conserved sequence “W-x3-I-x3-L-x2-R-x3-Q/E” (Figure 1B). At present, we have not yet clarified whether CgATG16 interacts with CgATG5. Furthermore, if CgATG16 and CgATG5 do indeed interact with each other, then what is the domain in CgATG16 that binds to CgATG5? Obviously, it is one of the core directions of our subsequent research to investigate the potential interaction mechanism between CgATG16 and CgATG5, which will help elucidate the molecular basis of CgATG16-mediated autophagy in *C. gloeosporioides*.

Previous studies on the function of ATG16 were mainly conducted in yeast and animal cells, focusing on its role as a component of ATG12-ATG5-ATG16 complex in regulating the formation of autophagosomes [24,33,34]. The functional characterization of ATG16 in pathogenic fungi was still limited, particularly in plant pathogenic fungi. AcATG16 of human pathogenic Acanthamoeba was confirmed to be involved in mitochondrial autophagy and encystation [12]. BbATG16, the homolog ATG16 of insect-pathogenic fungus *Beauveria bassiana*, contributed to asexual development, tolerance to oxidative stress, virulence and autophagosome formation [13]. In plant pathogenic fungus *Magnaporthe oryzae*, ATG16 (MoATG16) interacts with the PHD-domain protein Clp1 to regulate fungal development and virulence [14]. *Fusarium graminearum* FgATG16 indirectly regulated aerial hyphae development, asexual sporulation, deoxynivalenol (DON) synthesis, and virulence during wheat infection by maintaining the normal operation of the autophagy pathway [35]. In our study, we experimentally demonstrated the contribution of CgATG16 to mycelial growth rate and biomass (Figure 4), the development of the infection structure and primary invasion (Figure 5), which might be the mechanism by which ATG16 regulated pathogenicity of *C. gloeosporioides* to the rubber tree (Figure 3).

Fungal melanin is a complex secondary metabolite and has a variety of functional properties and biological activities [36]. In melanin-producing pathogenic fungi, melanin played a critical role in conidiation, appressorium development, cell wall integrity and in response to biotic and abiotic stresses [37,38,39], and also contributed to invasion through mediating the buildup of high pressure in the appressorium providing the essential driving force for mechanical penetration [40]. In our study, the melanin content of Δ*CgATG16* was significantly lower than that of WT (Figure 4D). Fungal melanin synthesis was catalyzed by a series of enzymes by melanin synthesis [41]. In *C. gloeosporioides*, polyketide synthase CgPKS1 and scytalone dehydratase CgSCD1 were demonstrated to be involved in melanin synthesis and pathogenicity [26,27]. Therefore, we investigated the transcriptional expression of *CgPKS1* and *CgSCD1* in WT, Δ*CgATG16* and Res-Δ*CgATG16*, and the results showed that the expression levels of *CgPKS1* and *CgSCD1* in Δ*CgATG16* were significantly lower than that in WT (Figure 4E), suggesting that *CgATG16* modulated melanin synthesis through CgPKS1 and CgSCD1. Considering that CgATG16 was required for efficient infection of *C. gloeosporioides* on rubber tree leaves and onion epidermal cells (Figure 3 and Figure 5F–G), we speculated that the regulation of CgATG16 on virulence may be related to melanin-mediated infection structure development and infection process.

As is well known, ATG16 plays a crucial role during autophagy. The formation of fungal functional appressorium requires the transfer of conidial cell contents through an autophagic recycling into the developing appressorium [42]. The appressorium formation process in *C. gloeosporioides* is accompanied by the occurrence of autophagy [17]. Studies in *M. oryzae* revealed that autophagy pathway is linked to appressorium morphogenesis, maturation, penetration and pathogenicity [3,43,44]. In the autophagy process, PEylation process of ATG8, the key step for autophagosome formation, was catalyzed by ATG16 complex [29]. The functional studies of these related ATG proteins in plant pathogenic fungi contribute to the understanding of the roles of autophagy in pathogenesis. In *M. oryzae*, deletion of MoATG5 and MoATG8 markedly disrupted autophagosome formation, conidiation, appressorium-mediated penetration, and pathogenicity [40]. Similarly, *C. orbiculare* CoATG8 and *C. scovillei* CsATG8 were required for autophagy occurrence, appressorium development and full virulence on host plants [41,42]. In our study, the absence of CgATG16 not only resulted in defective autophagy and appressorium development, but also resulted in impaired growth and sporulation (Figure 6 and Figure 7), suggesting the complex functions of ATG16 in the autophagy-mediated pathogenicity of plant pathogenic fungi.

Multiple signaling pathways were involved in appressorial formation and development in plant pathogenic fungi. In *Magnaporthe oryzae*, activated cAMP/PKA signaling and inactivated TOR signaling (TORoff) were required for appressorium formation and development. The cAMP/PKA signaling was required for appressorium formation initiation and activated the cAMP production by the adenylate cyclase Mac1 [45]. TOR signaling was mediated by TOR kinase and inactivated TOR kinase (TORoff) induced autophagy and appressorium formation [45,46]. In our study, exogenous addition of rapamycin partially restored the appressorial formation rate of *CgATG16* deletion strain (Figure 8A) and the phosphorylation level of S6K1 in *CgATG16* deletion strain was significantly increased (Figure 8C), indicating that CgATG16 might function on appressorium formation by modulating TOR activity. Our data also demonstrated that exogenous addition of cAMP restored the appressorial formation rate of *CgATG16* deletion strain (Figure 8B), indicating that cAMP signaling was also involved in CgATG16-mediated appressorial formation. Furthermore, we proved that the content of cAMP in *CgATG16* deletion strain was significantly lower than that in WT with decreased *CgMac1* gene expression (Figure 8D,E), suggesting that the cAMP synthesis mediated by CgMac1 was essential for CgATG16 mediated appressorial formation.

Taken together, our data revealed that CgATG16 was required for full pathogenicity of *C. gloeosporioides*. Given that CgATG16 was involved in mycelium growth, conidiation, appressorium formation, melanin production, and also required for mycelium autophagy and the autophagy during appressorium formation and development, we speculated that CgATG16 contributed significantly to pathogenicity by regulating the mycelium growth, melanin synthesis, invasion structure formation, and this process might be related to autophagy mediated by TOR and cAMP signaling. However, the mechanism by which CgATG16 regulates the TOR and cAMP signaling pathway remains to be further investigated.

## Data Availability

The original contributions presented in this study are included in the article/Supplementary Materials. Further inquiries can be directed to the corresponding author.

## Supplementary Materials

The following supporting information can be downloaded at: https://www.mdpi.com/article/10.3390/jof11120828/s1, Figure S1: Nucleotide sequence and amino acid protein sequence of CgATG16. Figure S2: Strategies and diagnosis for the *CgATG16* knockout mutants (Δ*CgATG16*) and complementary mutant (Res-Δ*CgATG16*). Figure S3: Lesion Phenotypes on *Hevea brasiliensis* Leaves Inoculated with *C. gloeosporioides* and Negative Control. Figure S4: Construction of GFP-ATG8/WT and GFP-ATG8/Δ*CgATG16* strains. Table S1: Primers are used in this study.
