# Supplementary material for: The CgATG16 Was Involved in Growth, Development and Virulence Through Autophagy Modulation in the Rubber Tree Anthracnose Fungus Colletotrichum gloeosporioides
Source: J Fungi (Basel). 2025 Nov 23;11(12):828. doi: 10.3390/jof11120828 (PMC12734049; doi:10.3390/jof11120828)
Supplement: Supplementary file 1 [file jof-11-00828-s001.zip › jof-3859445-supplementary.pdf]

Supplementary Table

Table S1. Primers are used in this study.

Primers	Sequence (5′→3′)
ATG16-3R	GGGCAACGTCGTCTTCCAG
ATG16-5F	ACCCGTTCCCCAGTGTTTC
ATG16-d3R	GTGTACGGCGAGCTAGGAG
ATG16-d5F	GAGCTGGATCTTGCGGTAC
ATG16-Sur-m3F	GAATTGCATGCTCTCACCGAGGCGGTCTATTTTT
ATG16-Sur-m3R	AAAAATAGACCGCCTCGGTGAGAGCATGCAATTC
ATG16-Sur-m5F	GCCAAAACCACCACAAAGTGCCAACGCCACAGTG
ATG16-Sur-m5R	CACTGTGGCGTTGGCACTTTGTGGTGGTTTTGGC
NP-ATG16-F(XbaI)	TCTAGAGCAGGGCGACCATACCCGTCT
ATG16-F（XbaI）	TCTAGAATGCCCGACTGGCGCACCGA
ATG16-R(BamHI)	GGATCCGCGCTCGTTTGCGAGGTTCA
GFP-5F(BamHⅠ)	GGATCCATGGTGAGCAAGGGCGAG
ATG8-R(SacⅠ)	GAGCTCTCACGCCGTCTCGAAACC
GPF-ATG8-MF	TGGACGAGCTGTACAAGATGCGATCCAAGTTCAA
GFP-ATG8-MR	TTGAACTTGGATCGCATCTTGTACAGCTCGTCCA
Sur-d3F	ACGAGGACCGCTACTCACATAC
Sur-d5R	GCGTTTGTAACTCTGCCTGTTTG
Sur-SLF	CCTCTGATATTGGAAGCGACGC
Sur-SLR	ATGTTGGCATAAGCCGAACCGT

Supplementary Figures

```
1     ATGCCCGACTGGCGCACCGAATACCTAGCCTCCTTCAGGGAGCAAGAGAAGAACAACCCA
1      M  P  D  W  R  T  E  Y  L  A  S  F  R  E  Q  E  K  N  N  P
61    GTCAACCTCGAAATCGTCCAGCTCTGCTCCGAACTGTCCGACCGCATCGCAGCCCTCGAA
21     V  N  L  E  I  V  Q  L  C  S  E  L  S  D  R  I  A  A  L  E
121   GCCGAAAAGGAGCTCCTCAAATCCAAAGTCGCCCCGAAAGCCTCTACCGCATCATCCACA
41     A  E  K  E  L  L  K  S  K  V  A  P  K  A  S  T  A  S  S  T
181   GGCAAGGAAGCGCCGCCGCAAATAGACACAATCAGCTCCGACCCGACCGTCACGCAGCTC
61     G  K  E  A  P  P  Q  I  D  T  I  S  S  D  P  T  V  T  Q  L
241   CAGCTCAACCTCGCCGAGGCGCTGCGCTCCAACGGCACGCTGCAGAGCCGCGCAAAGACG
81     Q  L  N  L  A  E  A  L  R  S  N  G  T  L  Q  S  R  A  K  T
301   GCCGAGGACGAGGCGCAGACGCTGCGCCGGAAGAACAGGGAGAACACGAAGCAGATCAAG
101    A  E  D  E  A  Q  T  L  R  R  K  N  R  E  N  T  K  Q  I  K
361   CAGCTGAGCGCCGAGAAGACGGCGCTGGCGACGAAGCTGCAGGATCGCGAGCACGAGCTG
121    Q  L  S  A  E  K  T  A  L  A  T  K  L  Q  D  R  E  H  E  L
421   CGCGAGAAGAGGAAGCTGGTTGAGAATGTGCAAGACGAAATGATCACGTTGAACCTCCAG
141    R  E  K  R  K  L  V  E  N  V  Q  D  E  M  I  T  L  N  L  Q
481   GTGGCCATGGCGGAGAAGGAGAGGGACAAGGTCAAGTCGGAAAACAAGGAGCTGGTCGAC
161    V  A  M  A  E  K  E  R  D  K  V  K  S  E  N  K  E  L  V  D
541   CGGTGGATGAAGAGAATGGCCCAAGAGGCTGACGCCATGAACCTCGCAAACGAGCGCTGA
181    R  W  M  K  R  M  A  Q  E  A  D  A  M  N  L  A  N  E  R  *
```

Figure S1. Nucleotide sequence and amino acid protein sequence of CgATG16. The underlined part is the amino acid sequence of the Autophagy-related protein 16 domain. * indicates a stop codon.

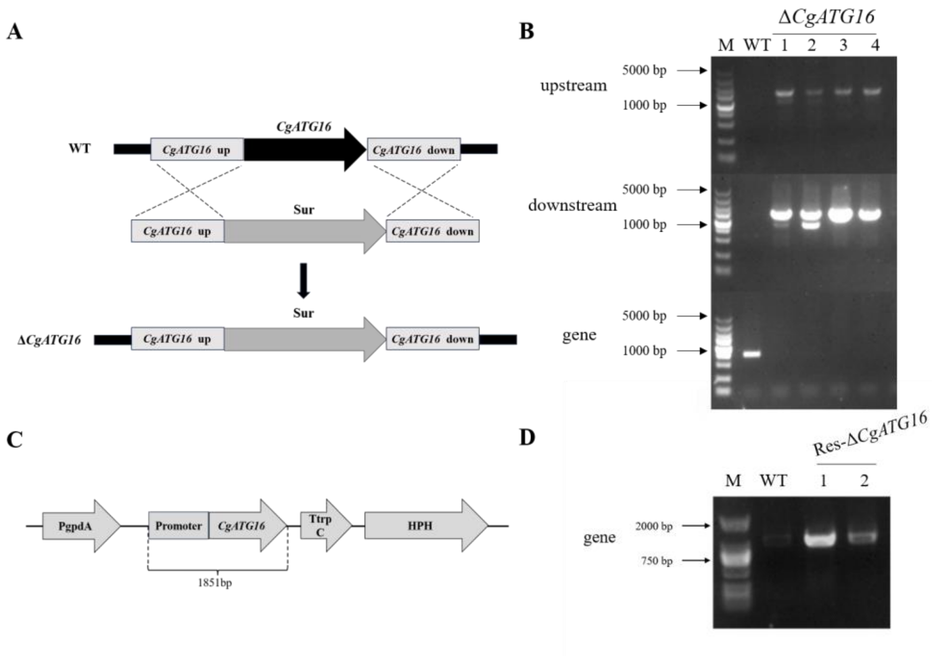

Figure S2. Strategies and diagnosis for the *CgATG16* knockout mutants(Δ*CgATG16*) and complementary mutant (Res-Δ*CgATG16*). (A) Principle of *CgATG16* knockout and complementation; (B) Detection of the upstream fragment, downstream fragment, and *CgATG16* gene in Δ*CgATG16*. (C) Principle of *CgATG16* complementation. (D) Detection of *CgATG16* gene in Res-Δ*CgATG16* strains. M: DL2000 DNA marker and DL5000 DNA；1-2: Different transformers.

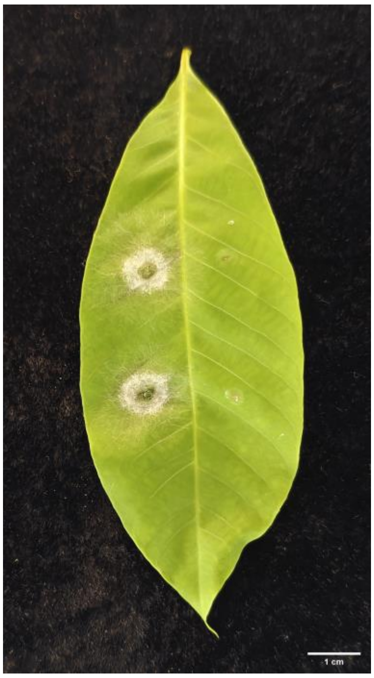

Figure S3. Lesions on *Hevea brasiliensis* leave inoculated with *C. gloeosporioides* and mock inoculation. Detached "light green" wounded leaves of *H. brasiliensis* cultivar 73-3-97 were incubated in a moist environment at 28°C under natural illumination, with observations made at 4 days post-inoculation (4 dpi).

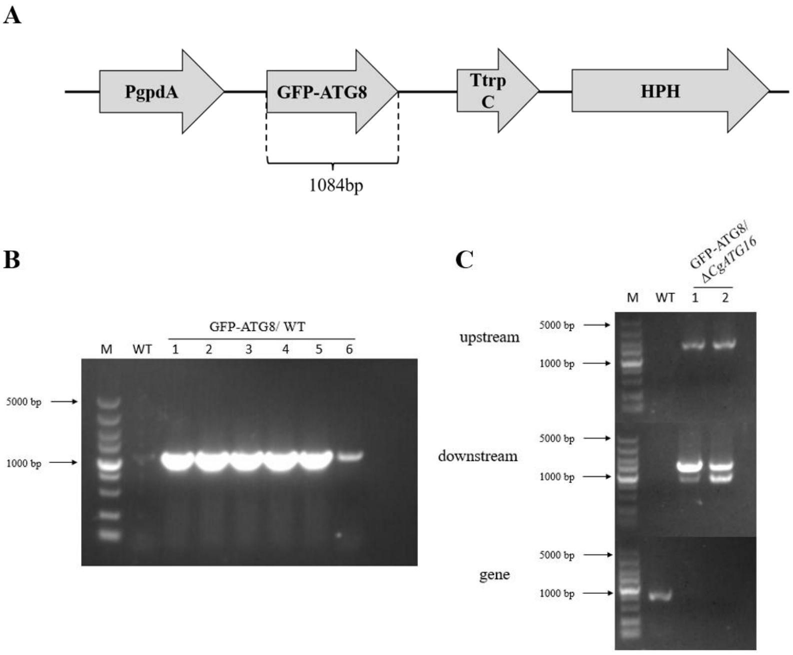

Figure S4. Construction of GFP-ATG8/WT and GFP-ATG8/Δ*CgATG16* strains. (A) The schematic diagram of GFP-ATG8 complementation. (B) Construction of GFP-ATG8/WT and detection of full-length GFP-ATG8. (C) Detection of the upstream fragment, downstream fragment, and *CgATG16* in GFP-ATG8/Δ*CgATG16*. M: DL5000 DNA marker; 1-6: Different transformers.
